# True Versus Sham Thread Embedding Acupuncture for Spasticity-Related Symptoms and Disabilities in Multiple Sclerosis: Study Protocol of a Randomized Controlled Trial

**DOI:** 10.3390/healthcare14203467

**Published:** 2026-10-15

**Authors:** Malika Vaez-Tehrani, Mohammad Ali Nahayati, Ehsan Mosa-Farkhani, Shirin Shafaei, Mohammad Nematshahi, Hamidreza Bahrami-Taghanaki

**Affiliations:** 1 Department of Acupuncture, School of Persian and Complementary Medicine, Mashhad University of Medical Sciences, Mashhad 9177899191, Iran; vaeztehrany@gmail.com (M.V.-T.); shafaeish4001@mums.ac.ir (S.S.); m.nematshahi.1975@gmail.com (M.N.); 2 Student Research Committee, School of Persian and Complementary Medicine, Mashhad University of Medical Sciences, Mashhad 9177899191, Iran; 3 Department of Neurology, School of Medicine, Mashhad University of Medical Sciences, Mashhad 9176699199, Iran; nahayami@ucmail.uc.edu; 4 Waddell Center for Multiple Sclerosis, Department of Neurology, University of Cincinnati, Cincinnati, OH 45219, USA; 5 Department of Epidemiology, School of Health, Mashhad University of Medical Sciences, Mashhad 9177943794, Iran; farkhanie@mums.ac.ir; 6 Social Determinants of Health Research Center, Mashhad University of Medical Sciences, Mashhad 9177943794, Iran; 7 Department of Anesthesiology, Faculty of Medicine, Sabzevar University of Medical Sciences, Sabzevar 9613873137, Iran

**Keywords:** multiple sclerosis, spasticity, spasticity-plus syndrome, gait, disability, acupuncture, thread embedding‌, polydioxanone, study protocol

## Abstract

**Introduction:** Multiple sclerosis (MS) is a chronic autoimmune disease and one of the most common causes of non-traumatic disability in young adults, with over 2.8 million individuals worldwide. Spasticity affects nearly 80% of people with MS (PwMS), contributing to pain, gait disturbance, bowel/bladder dysfunction, sleep/mood disorders, and impaired quality of life. The complex condition known as Spasticity-Plus syndrome is gaining attention. Current pharmacological treatments are limited by side effects and short duration of benefits. Thread Embedding Acupuncture (TEA), which involves embedding absorbable polydioxanone (PDO) sutures into acupoints, offers prolonged stimulation and has shown promise in managing spasticity, particularly after stroke. Several randomized controlled trials (RCTs) have reported positive effects in MS. This is a study that protocol aims to evaluate the efficacy and safety of True TEA (TTEA) compared with Sham TEA (STEA) in reducing spasticity and improving mobility and related symptoms among PwMS. **Methods:** This is a protocol of a randomized, sham-controlled, patient-, assessor- and analyst-blinded, superiority clinical trial. Thirty-eight eligible PwMS with moderate-to-severe spasticity will be randomized 1:1 to either TTEA or STEA. Both groups will undergo puncture at the same 30 acupoints but only the TTEA group will receive PDO thread implantation. The primary outcome is change in disability (EDSS). Secondary outcomes are Numeric Rating Scale for Spasticity (NRS-S), walking ability (MSWS-12), activities of daily living (Barthel Index), pain (McGill Pain Questionnaire), fatigue (MFIS), sleep quality (PSQI), urinary symptoms (ABSST), mood (HADS), quality of life (EQ-5D-5L), overall treatment satisfaction, willingness to repeat and recommendation to others. Assessments will be conducted at baseline, 4 weeks, and 12 weeks post-intervention. Intention-to-treat analysis will be performed. **Perspective:** To our knowledge this trial will be the first RCT to evaluate TEA in spasticity-related symptoms in PwMS. By using a sham-controlled design, it will determine whether TEA confers benefits beyond needle puncture alone. Findings may provide evidence for incorporating TEA as a complementary option in MS symptom management and inform future larger-scale trials. Trial registration: IRCT20121228011912N8 (22 March 2024; Updated: 13 July 2026).

## 1. Introduction

Multiple sclerosis (MS) is a chronic autoimmune demyelinating disease of the central nervous system and the leading cause of non-traumatic disability in young adults. Its prevalence exceeds 2.8 million individuals worldwide. A multidisciplinary approach is necessary for managing the unclear underlying pathophysiology and symptoms of irreversible axonal damage [1]. Spasticity is characterized by increased muscle tone, resulting from upper motor neuron damage. It occurs in about 80% of patients within five years of diagnosis and is closely associated with gait disturbance, pain, fatigue, sleep disruption, and bladder dysfunction, significantly impairing quality of life (QoL) [1,2,3,4]. Recently, the concept of Spasticity-Plus Syndrome was introduced to provide a more holistic framework for understanding spasticity and its associated symptom cluster [2]. Reducing excessive muscle tone can improve voluntary motor function and other related symptoms [5].

Current pharmacological therapies for spasticity, such as baclofen, tizanidine, and botulinum toxin, are often limited by side effects, high costs, short action duration, tolerance, and potential for immunity alteration [2,3,4,5,6]. Non-pharmacological interventions, including physiotherapy, electrical or magnetic stimulation, vibration, and acupuncture, have shown benefits but robust evidence remains insufficient [7,8]. Globally, many people with MS (PwMS) seek complementary and alternative medicine (CAM) for holistic care [9] (Supplementary Table S1 [10,11,12,13,14,15,16,17,18]). In the United States, approximately 20% of PwMS report using acupuncture [9].

Thread Embedding Acupuncture (TEA) is a relatively novel acupuncture modality in which absorbable sutures, such as polydioxanone (PDO), are implanted into selected acupoints [19]. The gradual absorption of these sutures provides prolonged mechanical and biochemical stimulation, offering stronger and longer-lasting effects than traditional acupuncture needles. This feature makes TEA particularly attractive for intractable conditions such as spasticity, where frequent treatment sessions may be beneficial but are often impractical for disabled patients [19,20]. Evidence suggests that TEA has neuromodulatory, anti-inflammatory, and regenerative effects, with clinical investigations in conditions such as post-stroke spasticity, cerebral palsy and epilepsy [9,20,21,22,23] (Supplementary Table S2 [22,23,24,25,26,27,28,29,30,31,32,33,34,35,36,37,38,39,40,41,42,43]). TEA demonstrates a generally favorable safety profile, with adverse events being typically mild and transient. Severe events are rare, particularly those associated with older catgut materials [19]. World Health Organization recognizes acupuncture as a CAM for post-stroke spasticity, while Chinese guidelines already recommend TEA [20,21]. Acupuncture may modulate the GABAergic system and mGluR1 and promote local release of neurotrophic factors such as BDNF and NGF, contributing to neural repair and remyelination [44,45] (Supplementary Table S3 [46,47,48,49,50,51,52,53,54,55,56,57,58,59,60,61,62]).

The American National MS Society recognizes acupuncture as a symptomatic treatment option [63]. Experimental and clinical studies verify that various modalities on acupoints may influence neuroimmunologic pathways and improve MS symptoms [9] (Supplementary Table S4 [4,7,8,9,64,65,66,67,68,69,70,71,72,73,74,75,76,77,78,79,80,81,82,83,84,85,86,87,88,89,90,91,92,93,94,95,96,97,98,99,100,101]). The results of a recent network meta-analysis of 23 Randomized Controlled Trials (RCTs) on the performance of acupoint stimulation therapy in conjunction with standard pharmacotherapy suggested that electroacupuncture combined with acupoint injection showed the greatest effectiveness in improving neurological function and activities of daily living, while acupressure therapy was most effective in alleviating fatigue [84]. Another meta-analysis of 11 RCTs showed that acupuncture, when combined with medication, is more effective in improving disability status and enhancing QoL compared to medication alone [85]. However, systematic reviews highlight that research on acupoints in MS, particularly regarding spasticity, has often heterogeneity and lacked methodological rigor, precluding definitive conclusions [7,8,9,84,85].

Given the high prevalence and management limitations of spasticity, and the growing reliance on CAM among PwMS, evaluation of TEA is warranted as part of integrative MS care.

This RCT is designed to investigate the efficacy and safety of true versus sham TEA in PwMS. For a multidimensional evaluation, the primary outcome is assessment of disability. Secondary outcomes include spasticity, walking, pain, and activities of daily living, fatigue, sleep, mood, urinary symptoms, QoL, and overall treatment satisfaction.

## 2. Methods

### 2.1. Study Description and Design

This study is a two-center, randomized, superiority, sham-controlled, patient-, assessor- and analyst-blinded clinical trial designed to evaluate the efficacy and safety of True TEA (TTEA) versus Sham TEA (STEA) in PwMS who are experiencing moderate to severe spasticity. A total of 38 eligible patients will be randomly assigned in a 1:1 ratio. The intervention consists of a single treatment session, with follow-up evaluations at 4 and 12 weeks post-intervention.

Participants will be recruited from the Neurology Clinic of Qaem Hospital, and interventions will be conducted at the Acupuncture Clinic of Imam Reza Hospital, both affiliated with Mashhad University of Medical Sciences (MUMS), Iran. The study protocol adheres to the Consolidated Standards of Reporting Trials (CONSORT) and the Standards for Reporting Interventions in Clinical Trials of Acupuncture (STRICTA) [102,103]. This report is written based on Standard Protocol Items: Recommendations for Interventional Trials (SPIRIT) checklist [104].

### 2.2. Eligibilty Criteria

#### 2.2.1. Inclusion Criteria

The participants must meet all the following criteria:

- A definitive diagnosis of MS according to the most current diagnostic standard, McDonald 2024 criteria [105] will be confirmed by a neurologist with an MS fellowship;
- Expanded Disability Status Scale (EDSS) score < 7 [106] to enroll ambulatory MS patients who can reliably complete mobility outcome measures, minimizing ceiling effects and confounding associated with wheelchair-dependent disability and related comorbidities;
- Moderate to severe spasticity is defined as a Numeric Rating Scale for Spasticity (NRS-S) score ≥ 4, with an inadequate response to usual antispasmodic medications [107];
- Age ≥ 18 years;
- Willingness to participate and provide written informed consent.

#### 2.2.2. Exclusion Criteria

Subjects who meet one of the following criteria will not be included in the study:

- Use of anticoagulants;
- History of coagulopathy;
- Acupuncture was given within the past 6 months;
- Any history of thread embedding;
- Changes in maintenance MS treatments or relapse and corticosteroid pulse therapy within the past 2 months;
- Allergy to Cephalosporins or any component of the thread;
- Presence of unstable or inadequately controlled comorbid diseases, active autoimmune diseases, other critical chronic systemic diseases, or cancers that may confound the clinical course, interfere with safety assessment, or affect study participation and outcome evaluation (clinically stable and well-controlled comorbidities such as hypertension and type 2 diabetes mellitus are not considered exclusionary);
- Pregnant or breastfeeding, or failure to use reliable contraception.

#### 2.2.3. Withdrawal Criteria

Subjects who fulfil any of the following criteria will be prohibited from continuing the study:

- Unwillingness to continue participation or withdrawal of consent;
- Allergic reactions or resistant bleeding requiring medical intervention post-procedure;
- MS relapse requiring corticosteroid pulse therapy during the study;
- Changing the maintenance drug.

### 2.3. Participant Recruitment

Potential participants will be identified through referrals from the MUMS Neurology Department and announcements in PwMS social media groups. Initial eligibility screening and study briefing will be conducted via phone. The next screening will be performed by an MS fellowship for an in-person neurological and spasticity evaluation. Eligible individuals will be referred to the acupuncture clinic. After confirming eligibility and providing comprehensive study information by acupuncture supervisor, participants will provide written informed consent prior to enrolment.

### 2.4. Arms and Interventions

#### 2.4.1. Randomization and Allocation Concealment

Randomization will be performed via block randomization with block sizes of 4 and 6, generated by a validated online randomization tool [108]. The randomization sequence will be sealed in waxed opaque, sequentially numbered envelopes managed by an independent Research Coordinator (RC1). Upon providing informed consent, the RC2 will assign participants to groups by opening the next envelope in sequence. Allocation information will be accessible only to the practitioner performing the intervention and RC2.

#### 2.4.2. Blinding

The participants will be blinded to group assignments. Both the TTEA and the STEA procedures will be performed by the same practitioner in identical settings. Sham TEA will mimic the true procedure using empty needles and guides without thread implantation to maintain blinding. The RC2 provides needles out of sight to patients. Patients with prior experience with thread embedding are excluded to reduce detection bias. To prevent communication between participants, appointments will be scheduled separately. While the acupuncturist cannot be blinded owing to the nature of the intervention, outcome assessors and data analysts will remain blinded to group allocation.

#### 2.4.3. Intervention and Control Groups

**Figure 1:**
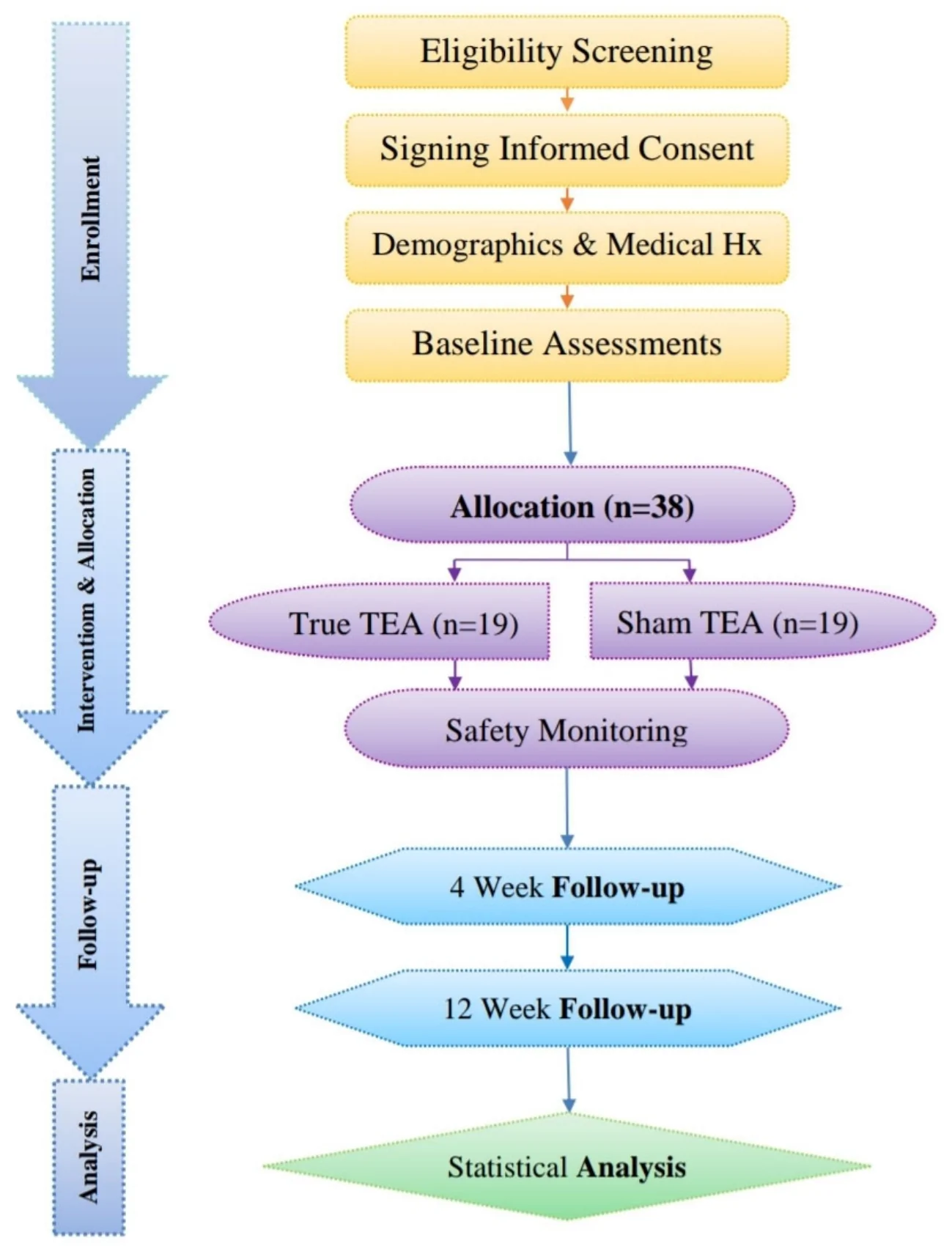
Study Flowchart Illustrating Participant Progression. This flowchart illustrates the key stages of the randomized controlled trial comparing True versus Sham Thread Embedding Acupuncture (TEA) in patients with multiple sclerosis and spasticity. It outlines the process from participant eligibility screening and basic assessments through randomization and intervention administration into true-intervention and sham-control groups, follow-up assessments at 4 and 12 weeks, and statistical analysis. The flowchart is designed in accordance with CONSORT guidelines to ensure transparency and clarity in reporting. Abbreviations: Medical Hx: Medical History.

The study includes enrolment, treatment, and follow-up phases (Figure 1, Table 1). If participants voluntarily signed the informed consent form, the investigator will record baseline demographic characteristics and medical, drug, concomitant treatment, and family history. Baseline assessment by the appropriate questionnaires will be performed.

#### 2.4.4. Acupoint Selection

Thirty acupoints were selected on the basis of clinical experience and previous studies [8]:

Yin–Yang balance: GV14 (Dazhui), GV20 (Baihui), CV4 (Guanyuan), and CV12 (Zhongwan)

Root causes of MS: BL18 (Ganshu), BL20 (Pishu), BL23 (Shenshu), SP6 (Sanyinjiao), SP9 (Yinlingquan), ST36 (Zusanli), KI3 (Tai Xi), GB20 (Fengchi), GB34 (Yanglingquan), and LI11 (Quchi)

Spasm and contraction control: LR3 (Taichong), LI4 (Hegu), and BL57 (Chengshan).

**Figure 2:**
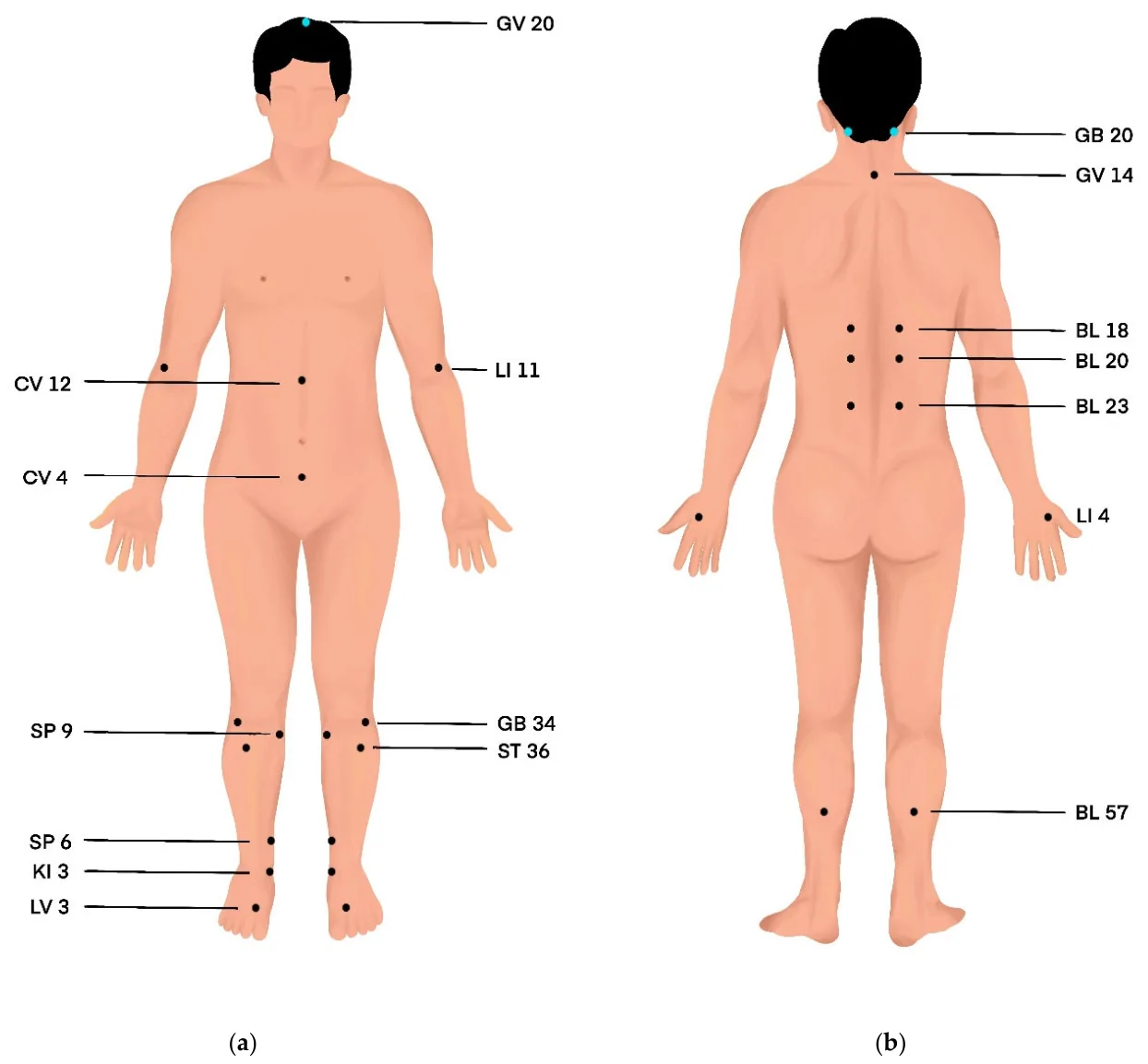
Acupoint Locations. Acupoint locations used in the true-intervention and sham-control groups of thread embedding acupuncture for Multiple Sclerosis patients with spasticity. (**a**) Posterior view of the back showing selected acupoints: GB20 (Fengchi), GV14 (Dazhui), BL18 (Ganshu), BL20 (Pishu), BL23 (Shenshu), and BL57 (Chengshan). (**b**) Anterior view of the front showing selected acupoints: GV20 (Baihui), CV4 (Guanyuan), CV12 (Zhongwan), LI4 (Hegu), LI11 (Quchi), SP6 (Sanyinjiao), SP9 (Yinlingquan), ST36 (Zusanli), KI3 (Tai Xi), LV3 (Taichong), and GB34 (Yanglingquan).

Patients will be positioned prone for intervention on GB20, GV14, BL18, BL20, BL23, and BL57 and supine for the remaining points (Figure 2).

#### 2.4.5. Procedure

After marking acupoints, wearing mask and sterile gloves, and skin sterilization with povidone-iodine, needles will be inserted at 90 degrees, except at BL18 and BL20, where tangential insertion minimizes the risk of pneumothorax.

**Figure 3:**
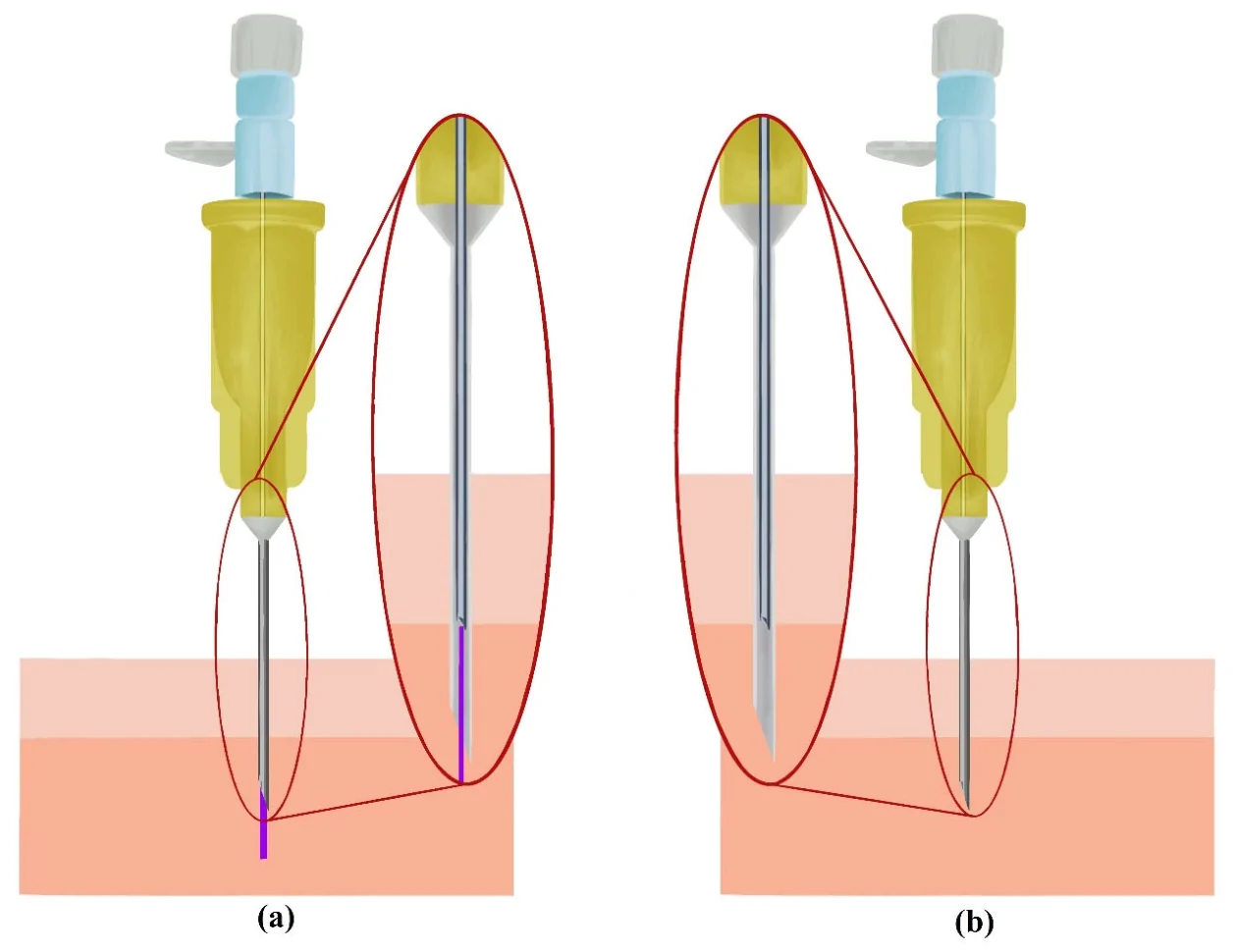
True versus Sham Thread Embedding Acupuncture Procedures. True-intervention and Sham-control procedures with thread embedding acupuncture. (**a**) True Thread Embedding Acupuncture (TTEA) procedure: Sterile polydioxanone (PDO) threads (Violet line) are implanted into acupoints via a 20-gauge needle guided by a 22-gauge angiocath, providing sustained stimulation. (**b**) Sham Thread Embedding Acupuncture (STEA) procedure: Identical needle insertion without thread implantation, serving as the control to mimic the intervention as a control for therapeutic effects. Both procedures are performed under sterile conditions with patients blinded to group allocation. This design enables the evaluation of the specific effects of thread embedding beyond needle puncture alone.

True TEA Group: Sterilized 2 cm 0 monofilament polydioxanone threads will be implanted via 20-gauge needles and a 22-gauge angiocath needle as a guide (Figure 3a).

Sham TEA Group: An identical procedure in which empty needles and guides without thread implantation will be injected (Figure 3b). This design is consistent with previous studies [23].

The interventions will be administered in a single session [19,109]. The thread-embedding procedure will be performed in accordance with the Chinese standard for thread-embedding therapy [110]. Further details of the intervention are described in the STRICTA checklist (Table 2) [103].

#### 2.4.6. Post-Trial Treatment

After study completion, participants in the control group may receive TEA treatment on the basis of clinical outcomes or for other acupuncture-responsive complaints upon patient request and medical evaluation.

### 2.5. Safety Measures and Adverse Events Management

To ensure participant safety and systematically record potential intervention-related risks, a multi-stage monitoring protocol has been established [19,43], categorized into immediate, intermediate, and long-term phases:

- Immediate Assessment (Early Phase): Participants will be closely observed for 30 min post-procedure in the clinical setting. This phase focuses on screening for immediate adverse events (AEs), such as vasovagal reactions, acute pain, or localized bleeding at the insertion sites.
- 48-h Follow-up (Intermediate Phase): A structured telephone interview will be conducted 48 h after the intervention. This step is specifically designed to screen for intermediate reactions, including delayed allergic responses to the PDO threads, localized inflammation, or any persistent discomfort.
- Emergency Hotline (Ongoing Phase): A dedicated 24/7 emergency contact number (hotline) is provided to all participants. This allows for the immediate reporting of any delayed AEs or unexpected health concerns throughout the entire follow-up period, ensuring a continuous safety net beyond the scheduled assessment.

All adverse events will be systematically recorded in the case report forms, including details regarding their type, severity, onset, duration, management, outcome, and potential relationship to the intervention.

Due to immunosuppression in patients, prophylactic oral cephalexin (500 mg every 6 h for 2 days) will be prescribed to prevent infection, in accordance with institutional protocols. Pain management includes cold compresses or Ibuprofen as needed.

PwMS are under close observation with laboratory monitoring to ensure the safe continuation of their medications. The most recent laboratory results will be reviewed by both the neurologist and the acupuncturist before the intervention.

Participants must maintain current MS immunotherapy and symptomatic treatments throughout the study, but the initiation of new therapies, including new anti-spasmodic agents, will exclude the subject.

Potential mild adverse events include sensitivity reactions, which can be managed with antihistamines, and minor bleeding, which can be controlled with a cold compress. In the case of severe adverse events, such as infections requiring antibiotics or massive bleeding, the researchers will provide appropriate treatment, and the participants will be withdrawn from the study.

If recurrent severe adverse events occur, stopping the trial will be considered.

### 2.6. Outcome Measures

**Figure 4:**
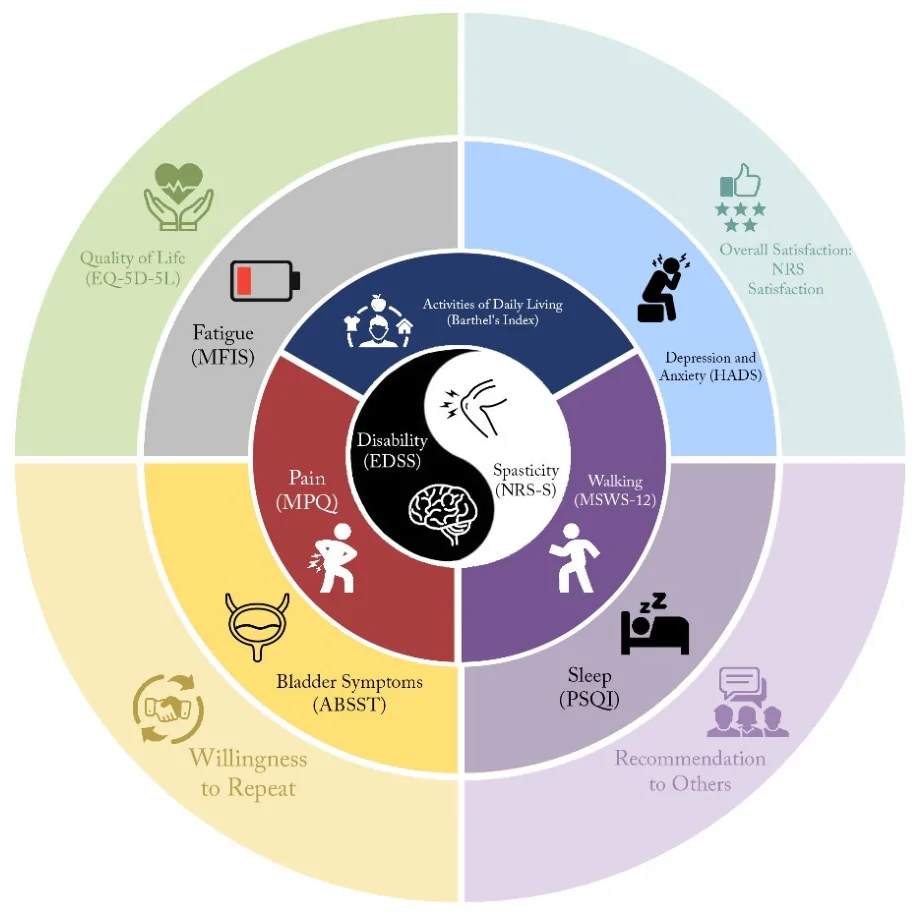
Concentric domains of outcome assessment in spasticity-plus syndrome. The center represents five core clinical outcomes, disability (EDSS), and spasticity severity (NRS-S), followed by pain (MPQ), walking ability (MSWS-12), and activities of daily living (Barthel’s Index). The outer domains include fatigue (MFIS), depression and anxiety (HADS), sleep quality (PSQI), bladder symptoms (ABSST), quality of life (EQ-5D-5L), satisfaction, recommendation to others, and willingness to repeat the intervention.

Assessments will be performed at baseline and at 4 and 12 weeks post-intervention. The baseline assessment will be conducted in person before the intervention, whereas follow-up assessments will be performed through structured telephone interviews. Validated Persian versions of all questionnaires will be used. The study outcomes are briefly presented in Figure 4. All these outcome measures are commonly used in MS research and have been widely applied in MS clinical trials [111,112,113].

#### 2.6.1. Primary Outcome

1. Expanded Disability Status Scale (EDSS): The main MS grading and physician-assessed scale (0–10: normal to death) measures neurological disability across eight functional systems, follow-up EDSS scores will be estimated based on patient-reported ambulatory status [106].

EDSS is the sole primary outcome and the only measure underlying the sample size calculation and statistical powering (Section 2.7).

#### 2.6.2. Secondary Outcomes

1. Numeric Rating Scale for Spasticity (NRS-S): Patient-reported spasticity severity on a 0–10 scale [107].
2. Multiple Sclerosis Walking Scale-12 (MSWS-12): Patient-reported 12-item 2-week-recall scale assessing walking ability and related challenges [114].
3. Barthel Index: This index measures independence in basic activities of daily living, such as feeding, dressing, and bladder and bowel control [115,116].
4. McGill Pain Questionnaire (MPQ): This tool assesses pain quality and intensity through descriptive word selection in 20 sections to provide quantitative measures of clinical pain [117,118].
5. Modified Fatigue Impact Scale (MFIS): Patient-reported 21-item 1-month-recall for assessing the impact of fatigue [119].
6. Pittsburgh Sleep Quality Index (PSQI): Patient-reported 19-item, 1-month recall questionnaire evaluating sleep quality [120,121].
7. Actionable Bladder Symptom Screening Tool (ABSST): Patient-reported 8-item 7-day recall questionnaire evaluating lower urinary tract symptoms and their daily impact [122].
8. Hospital Anxiety and Depression Scale (HADS): Patient-reported 14-item scale for 7-day recall assesses anxiety and depression symptoms [123,124].
9. Euro Quality of Life 5-Dimension 5-Level (EQ-5D-5L): Patient-reported questionnaire measures health-related quality of life across five domains [125,126].
10. Overall satisfaction: A 1–10 scale will be used to assess overall treatment outcomes, willingness to repeat the treatment, and likelihood of recommending the treatment to other patients.

### 2.7. Sample Size Calculation

On the basis of prior research [73], the EDSS was selected as the primary outcome for sample size calculation. Assuming an effect size of 0.8 and a standard deviation of 1.0, with a two-sided α = 0.05 and 80% power, 17 participants per group are required. Accounting for an anticipated 10% dropout rate, 19 participants per group (total *n* = 38) will be enrolled.

### 2.8. Statistical Analysis

The primary analysis will be conducted on the intention-to-treat (ITT) population, defined as all randomized participants with at least one post-baseline assessment of the primary outcome. A per-protocol (PP) sensitivity analysis will be conducted excluding participants with major protocol deviations.

A linear mixed-effects model (LMM) will be used as the primary analytical method for the repeated-measures primary outcome and for continuous secondary outcomes. The LMM will include intervention group (TTEA vs. STEA), time (Week 4, Week 12), and the group × time interaction as fixed effects, with baseline value as a covariate and participant as a random effect. This approach accommodates missing data under the missing-at-random (MAR) assumption without requiring complete-case exclusion. The primary covariance structure is pre-specified as unstructured (UN). Should convergence issues arise with the UN structure, a first-order autoregressive covariance structure will be used as the pre-specified alternative.

Patterns of missing data will be described. Sensitivity analyses using multiple imputation (MI) under MAR and missing-not-at-random (MNAR) assumptions will be conducted for the primary outcome if missingness exceeds 10%.

A pre-specified exploratory subgroup analysis stratified by MS clinical subtype (RRMS vs. progressive forms [primary/secondary]) will be conducted for the outcomes. Given the expected small subgroup sizes, these subgroup analyses will be considered exploratory and primarily descriptive; therefore, any findings, including formal interaction tests, will be interpreted with caution.

Analyses will be performed using Stata 16 (StataCorp, College Station, TX, USA). Results will be reported as estimated mean differences with 95% confidence intervals and two-sided *p*-values (<0.05 significant).

### 2.9. Withdrawal and Dropout

Participants may withdraw at any time without penalty. The reasons for dropout will be documented. Severe adverse events or protocol violations may prompt exclusion under the judgment of the principal investigator. Data from dropouts will be included in the intention-to-treat analysis where possible.

### 2.10. Investigators

The intervention will be conducted by a senior acupuncture specialist (MD, PhD in Acupuncture, Beijing University of Chinese Medicine, China) with 16 years of clinical experience in acupuncture and 13 years in TEA. The specialist will be responsible for acupoint localization and procedural oversight, with assistance from an MD and PhD candidate in Acupuncture.

### 2.11. Data Management

All participant data will be handled confidentially. Hardcopy data will be stored securely in locked cabinets; electronic data through double data entry will be password-protected and accessible only to authorized personnel. The principal supervisor will oversee data security. A blinded analyst will perform the statistical analyses, and all the results will be protected anonymously by a supervisor. Anonymized individual clinical data will be made available upon request from the corresponding author for research (non-commercial) purposes to individuals affiliated with academic or public health institutions.

### 2.12. Ethics

The study complies with the Helsinki Declaration and has received approval from the MUMS Ethics Committee (IR.MUMS.REC.1402.345, 12 March 2024). The trial is registered with the Iranian Registry of Clinical Trials (IRCT20121228011912N8, 22 March 2024, Update: 13 July 2026). Any protocol amendments require approval from the ethics committee and the registry. All enrolled subjects will be provided with a complete and comprehensive introduction, including the purpose, procedures, and possible risks of the study by acupuncture supervisor. The participants will have sufficient time to decide whether they wish to participate. Written informed consent, approved by the ethics committee, will be obtained from all participants before the start of the study.

### 2.13. Quality Control

Quality assurance will be ensured through independent oversight by a Trial Monitoring Committee, consisting of an external clinician-researcher, an independent statistician, and an external safety advisor, none of whom will be involved in the study. Monitoring will be conducted monthly during the first 3 months of recruitment or until 25% of the target sample has been enrolled, and quarterly thereafter. The committee will also perform ad hoc reviews within 7 days of any serious adverse event, major protocol deviation, or other safety concern. It will evaluate protocol adherence, recruitment progress, adverse event documentation, and data integrity. Serious adverse events will be reported in accordance with institutional and ethics committee requirements.

### 2.14. Dissemination Plans

The study findings will be published in peer-reviewed journals and presented at relevant scientific conferences. Furthermore, results will be uploaded in trial registry.

## 3. Discussion

This protocol describes the design of a randomized, sham-controlled trial to evaluate the effects of TEA on spasticity and related symptoms. To our knowledge, this is the first clinical trial to investigate TEA in MS. Currently, apart from our study, only 16 RCTs evaluating acupuncture in PwMS are registered internationally, with just one assessing spasticity and none utilizing TEA [127].

This study also incorporates rigorous methodological safeguards. The sham-controlled design [19,23] strengthens the ability to attribute outcomes specifically to TEA. The novelty lies in assessing whether embedding absorbable sutures provides additional and sustained benefits compared with just acupoints needle puncture (blood-letting), reported effective in previous studies [4].

The rationale for utilizing a single TEA session with PDO threads is based on the material’s degradation kinetics and the intended therapeutic mechanism. Unlike catgut, which degrades within 7–10 days and requires repeated applications [21], PDO sutures retain 74%, 50%, and 25% of their tensile strength at 2, 4, and 6 weeks, respectively, undergoing complete absorption only after 6–7 months [128]. The approximately 4-week structural half-life of PDO allows it to function as an implanted needle, providing continuous long-term acupoint stimulation that may approximate the effects of a conventional 12-session acupuncture course, thus making it a potentially suitable approach for neuromodulatory targets such as spasticity without requiring multiple invasive procedures. This single-session approach also reduces the treatment burden for PwMS with mobility limitations. To evaluate the short-term and sustained effects of this prolonged stimulation, follow-up assessments were scheduled at 4 weeks aligning with the active stimulation phase and 12 weeks to assess durability of the intervention, consistent with acupuncture protocols in MS [64,65,84,85] and previous trials demonstrating the efficacy and immune response of single-session PDO TEA [27].

By combining different measures this study aims to capture the foundational and multidimensional impact of TEA on MS in both biomedical and integrative medicine perspectives. This approach aligns with new concept of Spasticity-Plus Syndrome and holistic Traditional Chinese Medicine (TCM) principles, where improvement is not defined by a single symptom but by the restoration of overall function.

The primary clinical endpoint anchoring the main hypothesis and sample size calculation is the EDSS, the standard regulatory measure in Phase III MS trials. However, due to its non-linearity, subjective scoring and day-to-day variability, limited sensitivity to short-term change, and heavy weighting toward ambulation at scores (≥4.0), EDSS alone underestimates upper-limb function, pain, fatigue, mood, and patient-reported quality of life. Multidimensional batteries show significantly higher sensitivity to clinical change in MS than EDSS alone [29,30,31].

Accordingly, in line with the International Classification of Functioning, Disability and Health (ICF) framework and structured specialists’ discussion we selected a wide range of secondary outcome to better reflect the multidimensional burden of spasticity-plus syndrome [2,3,29,30,31]. In this context, the EDSS were the anchor and NRS-S were considered complementary core measure, capturing overall neurological disability and spasticity severity, respectively.

However, because the impact of spasticity extends beyond neurological examination and symptom intensity alone, additional assessments of pain, walking ability, and activities of daily living were included. Together, these patient-reported outcome measures provide a broader and clinically meaningful picture of spasticity-related disability, mobility restriction, and functional limitation.

The four key secondary outcomes (NRS-S, MSWS-12, Barthel Index, MPQ) will each be analyzed individually using the same mixed-effects model framework described; nominal *p*-values will additionally be reported after Holm–Bonferroni adjustment restricted to this secondary-outcome family, explicitly labeled as exploratory. This exploratory correction affects only the descriptive interpretation of secondary findings and does not alter the primary analysis or the enrollment target. A prespecified responder composite (defined as achieving the minimal clinically important difference in at least two secondary outcome domains without worsening any other measures) will also be reported descriptively, consistent with current trends in MS research [29,30,31].

A methodological limitation of this study is the telephone-based follow-up assessment of EDSS, as this scale is traditionally clinician-administered. Although no lower EDSS threshold was predefined, patients with clinically meaningful MS-related spasticity commonly have EDSS scores above 4.0, where the scale is largely determined by ambulatory status and assistance requirements. Because the upper inclusion criterion was EDSS < 7.0, structured telephone follow-up was considered feasible in most participants. However, patient-estimated maximum walking distance has shown only moderate agreement with objective clinical performance and may cross EDSS thresholds, while even clinical assessments may vary over short periods [129]. Therefore, EDSS findings should be interpreted together with the accompanying patient-reported outcomes.

The trial has several potential clinical implications. If effective, TEA could provide a minimally invasive and longer-lasting complementary therapy, reducing reliance on pharmacological options. Null or negative finding will prevent medical futility. Moreover, demonstration of TEA’s safety in an immunologically vulnerable population will provide reassurance for broader application such as surgical or cosmetic fields.

To ensure this safety profile in practice, we adhered to rigorous infection-control protocols. Specifically, the infection-control guidelines of Imam Reza Hospital (MUMS) recommend antibiotic prophylaxis for procedures involving more than 10 insertion points. This requirement was mandated by our institutional review board to mitigate the risk of localized infection in immunocompromised participants undergoing multi-site thread embedding. This precaution, combined with strict sterile technique, is expected to minimize infection risk.

Several challenges are anticipated. Recruitment may be limited by cultural unfamiliarity with acupuncture in regions like ours.

Regional conflicts and related disruptions during the recruitment period and follow-up may affect participant enrolment and could potentially influence the study. Blinding remains difficult in acupuncture research, although sham procedures, exclusion of prior TEA users, and blinding of assessor and analyst will minimize bias. As patients continue their standard treatments, confounding effects cannot be fully excluded, though this reflects real-world practice. The limited sample size may restrict the generalizability of the results, as the calculation based on the EDSS, may not fully power other outcomes, addressed in the statistical analysis.

Finally, although a standardized TEA protocol was applied to maximize internal validity and reproducibility, spasticity in MS is inherently heterogeneous and often warrants individualized treatment. Accordingly, a fixed acupoint protocol may not fully address the diversity of symptom patterns across patients, and this should be taken into account when interpreting the clinical applicability of the findings.

Despite the limitations, this study will provide controlled data on the efficacy and safety of TEA for MS-related spasticity and its functional consequences.

The results of this study may provide a foundation for larger multicenter trials with longer follow-up periods. Future studies may also incorporate biomarker-based assessments, such as inflammatory cytokines or electromyography, and examine whether individualized TEA protocols tailored to patients’ predominant symptom profiles provide additional therapeutic benefit over a fixed acupoint approach. More broadly, these findings may encourage further investigation of TEA in other neurological conditions characterized by spasticity or chronic disability.

## 4. Conclusions

This trial will be among the first and largest RCTs to comprehensively evaluate the efficacy and safety of TEA for MS-related spasticity symptoms. Its findings will provide valuable reference data for the integration of acupuncture modalities into multidisciplinary MS care and inform the design of future large-scale clinical trials.

## Figures and Tables

**Table 1:** SPIRIT Schedule. This SPIRIT (Standard Protocol Items: Recommendations for Interventional Trials) chart presents the detailed timeline of the trial activities, including participant enrollment, allocation, interventions (true and sham thread embedding acupuncture), and outcome assessments. The schedule specifies the timing of the baseline measurements, intervention sessions, and follow-up evaluations at 4 and 12 weeks.

	Study Period					
	Enrollment	Allocation	Post-Allocation			Post-Trial
Timepoint		0	Treatment (Day 0)	Follow-Up		Remedial True Treatment
				Week 4	Week 12	
**Enrollment**						
Eligibility screen	X					
Informed consent	X					
Demographics	X					
Medical, Drug, and Family Hx	X					
Allocation		X				
INTERVENTIONS:						
TTEA			X			
STEA			X			X
Safety Monitoring			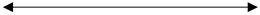			
ASSESSMENTS:						
Disability (EDSS)			X	X	X	
Spasticity (NRS-S)			X	X	X	
Walking (MSWS-12)			X	X	X	
Activities of Daily Living (BI)			X	X	X	
Pain (MPQ)			X	X	X	
Fatigue (MFIS)			X	X	X	
Sleep (PSQI)			X	X	X	
Bladder (ABSST)			X	X	X	
Depression and Anxiety (HADS)			X	X	X	
QoL (EQ-5D-5L)			X	X	X	
Overall Satisfaction				X	X	
Adverse Events			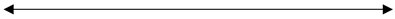			

Abbreviations: ABSST: Actionable Bladder Symptom Screening Tool, BI: Barthel Index; EDSS: Expanded Disability Status Scale, EQ-5D-5L: Euro Quality of Life 5-Dimension 5-Level, HADS: Hospital Anxiety and Depression Scale, Hx: History; MFIS: Modified Fatigue Impact Scale, MPQ: McGill Pain Questionnaire, MSWS-12: Multiple Sclerosis Walking Scale-12, NRS-S: Numeric Rating Scale for Spasticity, PSQI: Pittsburgh Sleep Quality Index, STEA: Sham Thread Embedding Acupuncture, TTEA: True Thread Embedding Acupuncture.

**Table 2:** STRICTA Checklist. This table outlines the essential components of the thread embedding acupuncture (TEA) intervention according to the Standards for Reporting Interventions in Clinical Trials of Acupuncture (STRICTA) guidelines. It ensures transparent and comprehensive reporting of both true-intervention and sham-control procedures, facilitating reproducibility and critical appraisal of the study.

Item	Description
Acupuncture rationale	Thread Embedding Acupuncture (TEA) for MS spasticity based on TCM theory, clinical experience, and literature.
Details of needling	30 acupoints, single session, sterile 0-size polydioxanone thread (2 cm), 20 G needle, 22 G angiocath guide.
Treatment regimen	One session, all acupoints treated in a single visit.
Other components	Standard care continued. Prophylactic antibiotics (cephalexin), pain management as needed.
Practitioner background	An academic professor in acupuncture, expert in TEA, assisted by a physician-acupuncturist
Control intervention	Sham TEA: identical procedure, empty needles/guides, no thread embedded.

## Data Availability

Anonymized individual clinical data will be made available upon request from the corresponding author (Bahramihr@mums.ac.ir) for research (non-commercial) purposes to individuals affiliated with academic or public health institutions.

## Supplementary Materials

The following supporting information can be downloaded at: https://www.mdpi.com/article/10.3390/healthcare14203467/s1, Table S1. Epidemiological Studies of Acupuncture Use in MS; Table S2. An Overview of Thread Embedding Acupuncture Studies; Table S3. Acupuncture Experimental Studies in Spasticity; Table S4. Acupuncture Studies in Multiple Sclerosis.

## Abbreviations

MS	Multiple Sclerosis
RCT	Randomized Controlled Trial
TCM	Traditional Chinese Medicine
CAM	Complementary and Alternative Medicine
GABA	Gamma-Aminobutyric Acid
BDNF	Brain-Derived Neurotrophic Factor
NGF	Nerve Growth Factor
PDO	Polydioxanone
TEA	Thread Embedding Acupuncture
TTEA	True Thread Embedding Acupuncture
STEA	Sham Thread Embedding Acupuncture
MUMS	Mashhad University of Medical Sciences
IRCT	Iranian Registry of Clinical Trials
RC	Research Coordinator
CONSORT	Consolidated Standards of Reporting Trials
STRICTA	Standards for Reporting Interventions in Clinical Trials of Acupuncture
SPIRIT	Standard Protocol Items: Recommendations for Interventional Trials
EDSS	Expanded Disability Status Scale
NRS-S	Numeric Rating Scale for Spasticity
MPQ	McGill Pain Questionnaire
ABSST	Actionable Bladder Symptom Screening Tool
MFIS	Modified Fatigue Impact Scale
PSQI	Pittsburgh Sleep Quality Index
HADS	Hospital Anxiety and Depression Scale
EQ-5D-5L	Euro Quality of Life 5-Dimension 5-Level
PROM	Patient-reported outcome measures
ICF	International Classification of Functioning, Disability and Health
