# Supplementary material for: True Versus Sham Thread Embedding Acupuncture for Spasticity-Related Symptoms and Disabilities in Multiple Sclerosis: Study Protocol of a Randomized Controlled Trial
Source: Healthcare (Basel). 2026 Oct 15;14(20):3467. doi: 10.3390/healthcare14203467 (PMC13704609; doi:10.3390/healthcare14203467)
Supplement: Supplementary file 1 [file healthcare-14-03467-s001.zip › SuppTables.TEA.MS-1.pdf]

Supplementary Table S1. Epidemiological Studies of Acupuncture Use in MS

Authors (Year, Country)	Study Design	Subjects (Number & Type)	Results Summary
Lotfi et al. (2024, Morocco) [10]	Quantitative Descriptive	98 PwMS	10.2% of cohort used acupuncture; T&CM positively perceived; T&CM use higher in progressive MS and financial barriers to pharma therapy.
Huybregts et al. (2018, Belgium) [11]	Retrospective descriptive	99 PwMS with motoric or psychosocial symptoms	44% used T&CM (most frequently homeopathy, acupuncture); T&CM users less satisfied than conventional treatment users.
Gotta et al. (2018, Germany) [12]	Structured questionnaire	343 PwMS	81.9 % used CAM for coexisting symptoms; 10-15% used Acupuncture
Kim et al. (2018, USA) [13]	Prospective clinical	524 PwMS, 304 healthy controls	CAM use similar in MS and controls; acupuncture among most used CAM for symptom relief and pain in MS.
Skovgaard et al. (2012, Nordic countries) [14]	Cross-sectional survey (questionnaire)	6455 MS society members	Wide CAM use (acupuncture among common modalities); CAM mostly used to improve general health and muscle strength; combined with conventional treatments.
Stoll et al. (2012, USA) [15]	Cross-sectional survey	111 PwMS (RRMS and progressive)	57.7% used CAM including acupuncture; CAM users reported mostly mild to moderate disability
Olsen (2009, USA) [16]	Literature review	PwMS in various surveys	CAM widely used (27–100%); acupuncture common; main targets pain, fatigue, stress.
Esmonde & Long (2008, UK) [17]	Mixed methods (questionnaire + workshop)	PwMS attending MS Trust Congress	Acupuncture rated as “extremely helpful” by 25–40%; users report symptom relief; research prioritized for CAM therapies.
Nayak et al. (2003, USA) [18]	Large cross-sectional survey	3,140 PwMS	57.1% used CAM including acupuncture; usage associated with dissatisfaction with conventional care; no efficacy data.

Supplementary Table S1. This table presents studies about prevalence of acupuncture use in PwMS.

Abbreviations (Alphabetically): CAM: Complementary and Alternative Medicine; PwMS: People with MS; T&CM: Traditional & Complementary Medicine

Supplementary Table S2. An Overview of Thread Embedding Acupuncture Studies

Authors (Year, Country)	Medical Condition	Study Design	Subjects	Outcomes	Results Summary
**TEA in Neurological Conditions**					
Li M (2025, China) [24]	Stroke	RCT	108 stroke patients; TEA treatment group (52), control (56)	levels of inflammatory markers, macrophage efferocytosis-related factors, NIHS, therapeutic efficacy, and adverse events	Significant NIHSS improvement in treatment group; altered serum SIRT1, HIF-1α, macrophage efferocytosis-related factors.
Xing BF et al. (2019, China) [25].	Stage I post-stroke shoulder-hand syndrome	RCT	60 patients, TEA + rehab vs acupuncture + rehab	VAS, FMA, hand edema score	TEA group had better VAS, hand edema, and Fugl-Meyer scores immediately and 1 month after treatment.
You M et al. (2024, China) [26]	Alzheimer's disease-like symptoms	Experimental animal	18 rats, divided into control, model, and TEA groups (6 each)	Morris Water Maze (learning and memory), histological brain tissue examination	TEA improved learning and memory in rats; histological brain tissue improvement suggesting potential for AD treatment.
Yang SQ et al. (2021, China) [27]	Alzheimer's disease	RCT	60 mild-moderate patients, observation (TEA + donepezil) vs control (donepezil alone)	MMSE, ADAS-Cog, ADL, NPI, Serum levels of APP and Aβ1-42	Combined group showed better cognitive scores and lower serum APP and Aβ1-42 than controls.
Zhou YL et al. (2008, China) [28]	Alzheimer's disease	Controlled study with fMRI	26 mild-moderate patients, TEA group (13) vs sham control (13)	fMRI, MMSE, ADAS-Cog	TEA improved cognitive scores (MMSE, ADAS-Cog) and activated cognition-related brain regions on fMRI compared to control.
Tao L et al. (2024, China) [29]	Migraine without aura	RCT	105 migraine patients, 3 groups (combination, TE, western med)	Headache attacks (Days, frequency, severity [VAS]), MSQ, safety	Combination therapy showed greater reductions in headache days, frequency, severity, and better QOL scores than others.
Jin YJ & Ge GL (2023, China) [30]	Tic disorders in children	RCT	68 children with tic disorders, observation group (34), control (34)	YGTSS, Serum level of NSE	TEA reduced YGTSS scores and serum NSE levels more than control; higher effective rate (87.9% vs 76.7%).
Nguyen VD et al. (2023, China) [23]	Drug-resistant epilepsy	RCT	54 patients, TEA (27) vs sham TEA (27)	Satisfactory seizure control, seizure freedom, heart rate and heart rate variability	TEA group had significantly better seizure control and heart rate variability improvements than sham TEA.
Ko MJ et al. (2023, Korea) [31]	Facial nerve palsy sequelae	Retrospective observational	82 patients treated with TEA	FDI, SFGS	Significant improvement in facial grading and disability index; no serious adverse events reported.
Jin BX et al. (2019, China) [22]	Ataxia children with cerebral palsy	RCT	70 ataxic CP children, TEA + rehab vs rehab	LSS, incurvation reflex, GMFM	TEA group showed enhanced sitting ability, reflex suppression, and higher overall response rate.
Wang TR et al. (2021, China) [32]	Hemifacial spasm	Literature review	-		Needle-embedding treatment methods summarized; need for further research on mechanisms and stage differentiation noted.
**TEA in Symptomatic Managements**					
Choi SJ (2026, Korea) [33]	Overactive bladder in postmenopausal women	RCT	68 postmenopausal women	Bladder diary, OABSS, KHQ, Adverse events	Both TEA and sham TEA/manual acupuncture improved symptoms over time, but there was no significant between-group difference in the primary or secondary outcomes. Nighttime voiding remained significantly reduced from baseline only in the TEA group at follow-up, but the between-group difference was still not significant. No serious adverse events were reported.

Choi SJ (2024, Korea) [34]	Overactive bladder in postmenopausal women	Prospective single-arm before-after	20 postmenopausal women	Bladder diary, OABSS, KHQ, Adverse events	Significant decrease in daily micturition frequency, OAB symptoms improved; no serious AEs reported.
Li XR et al. (2020, China) [35]	Insomnia	RCT	68 insomnia patients, TEA vs acupuncture	PSQI, SAS, SDS, TCM syndrome scores	Both effective; TEA showed better improvement in sleep quality and lowered medical costs.
Winarno EP et al. (2024, Indonesia) [36]	Myofascial pain in upper trapezius muscle	RCT	44 subjects, TEA group (22) vs sham TEA (22)	Pain score, NDI, PPT	TEA significantly reduced pain and disability at 4 and 8 weeks vs sham; increased pressure pain threshold.
Lee YJ et al. (2025, Korea) [37]	Knee osteoarthritis post-op pain	RCT	12 KOA patients with post-op pain, TEA+EA and Control (6 each)	VAS, WOMAC, EQ-5D, rescue medication consumption; adverse events	TEA+EA showed significant pain VAS improvement weeks 4, 6, 8; better WOMAC scores; no adverse events reported.
Goo B et al. (2022, Korea) [38]	Lumbar herniated intervertebral disc	RCT	70 patients, TEA vs sham TEA (1:1)	Pain, radiating pain, ODI, RMD, EQ 5-D, and global perceived effect	TEA showed no immediate difference; long-term follow-up showed improvement in low back pain and tendency for better function.
Pham TL et al. (2025, China) [39]	Low back pain (Non-specific chronic)	RCT	168 patients, 4 groups (42 each)	ODI, pain score, paracetamol use, QoL, global improvement, adverse events	TEA+ Auricular Acupuncture (AA) and TEA+sham AA groups showed greater improvement vs sham; TEA had sustained benefits; mild self-limiting AEs.
Kim JI et al. (2021, Korea) [40]	Neck pain (Non-specific chronic)	RCT	128 patients, TEA group vs physical therapy group	NPDS, MCID, Cervical spinal angle, BDI-II, BAI, PGIC, EQ-5D	TEA showed significant improvement in neck pain and disability, depression, anxiety, and quality of life over 13 weeks.
Kim E et al. (2019, Korea) [41]	Neck pain (Non-specific chronic)	RCT	106 patients, TEA + usual care vs usual care alone	NPDS, Clinical relevance (MCID), PPT, HADS, EQ-5D, PGIC	TEA + usual care showed significant improvements in Neck Pain and Disability Scale, anxiety/depression, QoL; safe with mild transient stiffness.
Sung WS et al. (2020, Korea) [42]	Chronic low back pain	RCT	38 patients, TEA + acupuncture vs acupuncture only	VAS, clinically relevant improvement (MCID), RMD, EQ-5D, PGIC, economic analysis, credibility test, safety assessment	TEA combined with acupuncture showed significant pain reduction and better global impression; no serious adverse events.
**Adverse Events of TEA**					
Ha S et al. (2024, Korea) [43]	Safety of TEA	Multicenter prospective observational pilot study	100 TEA patients	Adverse events	12 Adverse events (mostly mild local reactions); not serious; favorable safety profile confirmed.

Supplementary Table S2. This table presents studies about Thread Embedding Acupuncture (TEA) in various neurological conditions and a wide range of symptoms.

Abbreviations (Alphabetically): *AA: Auricular Acupuncture; ADAS-Cog: Alzhemer's Disease Assessment Scale-Cognitive section; ADL: Activity of Daily Living; BAI: Beck Anxiety Inventory; BDI-II: Beck Depression Inventory II; EA: Electroacupuncture; EQ-5D: European Quality of life- 5 Dimension; FDI: Facial Disability Index; FMA: Fugl-Meyer Assessment; GMFM: Gross Motor Function Measure; HADS: Hospital Anxiety and Depression Scale; KHQ: King's Health Questionnaire; LSS: Level of sitting scale; MCID: Minimal Clinically Important Difference; MMSE: Mini-Mental State Examination; MSQ: Migraine Specific Quality of life; NDI: Neck Disability Index; NIHSS: National Institutes of Health Stroke Scale; NPDS: Neck Pain and Disability Scale; NPI: Neuropsychiatric Inventory; NSE: Neuron-Specific Enolase; OABSS: Overactive Bladder Symptom Score; ODI: Oswestry Disability Index; PGIC: Patient Global Impression of Change; PPT: Pressure Pain Threshold; PSQI: Pittsburgh Sleep Quality Index; QoL: Quality of Life; RCT: Randomised Controlled Trial; RMD: Roland and Morris Disability questionnaire; SAS: Self-rating Anxiety Scale; SDS: Self-rating Depression Scale; SFGS: Sunnybrook Facial Grading System; TEA: Thread Embedding Acupuncture; VAS: Visual Analog ScalE, WOMAC: Western Ontario and McMaster Universities Arthritis Index; YGTSS: Yale global tic severity scale*

Supplementary Table S3. Acupuncture Experimental Studies in Spasticity

Authors (Year, Country)	Study Design	Subjects	Results
Ma LH et al. (2026, China) [46]	p-MCAO rat model	Sprague-Dawley rats with spastic hemiplegia	Acupuncture reduced spasticity, improved motor recovery, activated spinal PI3K/Akt signaling, promoted microglial M2 polarization, decreased IL-6 and TNF-α, increased IL-10 and TGF-β, improved H-reflex, and restored excitatory/inhibitory balance in the spinal cord.
He JL et al. (2025, China) [47]	MCAO rat model	Male rats with spastic hemiplegia	Acupuncture significantly reduced spasticity and improved motor performance, decreased spinal ventral horn hyperexcitability, downregulated NMDAR1, restored KCC2 expression/function, and acted through inhibition of the NMDAR-PP1/Calpain1-KCC2 pathway.
Qiongshuai Zh et al. (2025, China) [48]	MCAO rat model	32 rats; Control, Sham, Model, and Acupuncture groups	Acupuncture reduced infarct area, improved neurological deficits, decreased brain injury and apoptosis, lowered serum IL-1β, IL-6, and TNF-α, altered cell-type proportions in the ischemic penumbra, and identified 207 differential genes, with S100a8 and LST1 as key candidate genes linked to inflammatory regulation.
Zhang QY et al. (2025, China) [49]	MCAO rat model	Rats with post-stroke spasticity	Motion-Style Scalp Acupuncture (MSSA) improved neurological function, reduced muscle tone and spinal hyperreflexia, increased cerebral blood flow, reduced infarct area, and upregulated spinal 5-HT2AR and KCC2; effects were mediated through 5-HT2AR-dependent KCC2 reactivation.
Zhang Z et al. (2025, China) [50]	MCAO rat model	Rats with post-stroke spasticity	Waggle needling acupuncture modulated Glu/GABA-Gln cycle, reduced spasticity, improved motor behavior via GLT-1.
Kang X et al. (2025, China) [51]	MCAO rat model	Rats with post-stroke spasticity	Head acupuncture regulated GABAergic system and Wnt/β-catenin pathway, improved neurological function and muscle tone.
Zhang QY et al. (2023, China) [52]	rat model	36 rats in sham, stroke model, MSSA groups	Motion-style scalp acupuncture (MSSA) reduced spasticity, improved nerve function and H-reflex parameters in spastic rats.
Zhang Y et al. (2023, China) [53]	MCAO rat model	90 male rats in 6 groups	"Tiaoshen Tongluo" acupuncture improved neurological deficits and muscle spasticity via Nrf2/ROS signaling pathway; effects blocked by Nrf2 inhibitor.
Zhou Z et al. (2023, China) [54]	MCAO spastic paresis rats	Rats divided into control, sham, model, WN, PN groups	Waggle Needling (WN) at GB34 improved cerebral blood flow, neurological deficit scores, and spasticity better than Perpendicular Needling (PN); upregulated GABA receptor subunits.
Sun TY et al. (2022, China) [55]	MCAO rat model	MCAO rats vs sham	Acupuncture (particularly waggle needling) improved spastic muscle structure and motor function by enhancing GABA, KCC2, and GABAAγ2 in brainstem; waggle needling superior.
Qian X et al. (2022, China) [56]	MCAO rat model	48 rats in sham, model, and acupuncture groups	Acupuncture at GB34 + GV20 improved spasticity by upregulating Na+/K+-ATPase and glutamate transporters (EAATs) in hippocampus; combined stimulation superior.
Mu JD et al. (2022, China) [57]	MCAO rat model	Post-ischemic stroke rats	Acupuncture alleviated spinal hyperreflexia and motor dysfunction by activating KCC2-mediated spinal GABAA pathway; benefits reversed by KCC2 antagonist.
Li J et al. (2022, China) [58]	MCAO rat model	42 Wistar rats	EA at GV26 reduced phosphatidylinositol system overexpression, improved cerebral blood flow, and neurologic scores in acute cerebral infarction model.
Liu J et al. (2021, China) [59]	MCAO rat model	33 stroke rats	EA improved limb spasm and motor neuron excitability via regulating glutamate and GABA expression after stroke.
Xu YL et al. (2020, China) [60]	Animal study	Wistar rats with cerebral infarction	EA at Shuigou (GV26) upregulated sGC, PKG, and cGMP expression in cerebral artery smooth muscle, improving vascular function post-infarction.
Wang JX et al. (2021, China) [61]	MCAO rat model	MCAO rats	Waggle needling reduced spasticity in rats by activating KCC2-GABA(A) pathway; antagonist blocked effects, supporting mechanism role.
Wang JX et al. (2020, China) [62]	Animal study	Post-stroke spasticity rat model	Waggle needling reduced neurological deficits and muscle tone, decreased cerebral infarct volume, and modulated GABAT and GABA in cortex; effect comparable to baclofen

Supplementary Table S3. This table presents animal & mechanistic lab research investigating acupumcture effects in spasticity.

Abbreviations (Alphabetically): *EA: Electroacupuncture; MCAO: Middle Cerebral Artery Occlusion; MSSA: Motion-Style Scalp Acupuncture; p-MCAO: permanent Middle Cerebral Artery Occlusion; PN: Perpendicular Needling; WN: Waggle Needling*

Supplementary Table S4. Acupuncture Studies in Multiple Sclerosis

Authors (Year, Country)	Study Design	Subjects	Results Summary
**Clinical Studies**			
Khodaie et al. (2025, Iran/China) [64]	RCT	62 RRMS patients (31 acupuncture, 31 sham control)	Acupuncture improved cognitive functions (BICAMS), reduced fatigue, improved sleep quality, and reduced psychiatric symptoms vs control.
Khodaie et al. (2023, Iran/China) [65]	RCT	60 RRMS patients with fatigue (30 acupuncture + amantadine, 30 amantadine alone)	Acupuncture + amantadine reduced fatigue severity more than amantadine alone; improved quality of life; no adverse events reported.
Karpatkin et al. (2023, USA) [66]	Randomized Crossover Pilot	12 MS patients (7 completed all phases)	No significant changes in gait or balance; small significant increase in upper extremity strength; no adverse effects.
Lynning et al. (2021, Denmark) [67]	Single-blind RCT	66 MS patients randomized to real acupuncture, sham, or reference groups	No significant differences in cytokine plasma levels or quality of life after 4 weeks between groups.
Wang et al. (2017, China) [68]	RCT	42 RRMS patients (21 acupuncture, 21 shallow needling control)	Acupuncture reduced EDSS scores at 3 & 6 months, lowered annual relapse rates, and prolonged relapse interval vs control over 2 years.
Petrovics & Ondrejkovicova (2017, Slovakia) [69]	Case report	One female MS patient	197 acupuncture sessions with herbal supplements improved mobility, vocal cord spasms, articulation, pain relief, and fatigue.
Criado et al. (2017, Portugal/Germany) [4]	Cross-Over Controlled trial	20 RRMS patients	True acupuncture led to 95% improvement in 25-foot walk test versus 45% in sham acupuncture, indicating gait improvement.
Becker et al. (2017, USA) [70]	Pre/post nonexperimental pilot	14 women with MS	Acupuncture combined with health promotion reduced fatigue, stress, pain, depression, anxiety, sleep interference; improved quality of life and health-promoting behaviors.
Hao et al. (2013, USA) [71]	Case report	One 65-year-old male with 20-year MS history	Chinese scalp acupuncture with 16 treatments improved motor, sensory symptoms, balance, incontinence, and quality of life; patient in remission 26 months.
Foroughipour et al. (2013, Iran) [72]	Before-and-after clinical trial	40 MS patients with fatigue	Acupuncture combined with amantadine reduced fatigue severity in patients resistant to amantadine alone; significant improvement in Fatigue Severity Scale scores.
Quispe-Cabanillas et al. (2012, Brazil) [73]	Randomized patient- and evaluator-blind	31 RRMS patients on immunomodulators	Electroacupuncture significantly improved quality of life, including pain and depression reduction, compared to sham control.
Kopsky & Hesselink (2012, Netherlands) [74]	Case report	One MS patient with chronic central neuropathic pain	Acupuncture partially alleviated pain; combined with PPAR-α agonist palmitoylethanolamide, pain relief improved.
Tjon Eng Soe et al. (2009, Netherlands) [75]	Case series	MS patients with bladder dysfunction	Electroacupuncture reduced bladder dysfunction symptoms.
Donnellan & Shanley (2008, UK) [76]	Preliminary single-blind RCT	14 patients with secondary progressive MS	Minimal acupuncture showed greater psychological QoL improvement than Chinese medical acupuncture; no other significant differences; safe.
Gibson & Gibson (1999, Scotland) [77]	Pilot + RCT	61 MS patients	Neural therapy (modified acupuncture with local anesthetic injection) improved disability scores in 65-76%; safe and effective in study.
Steinberger (1986, Unknown) [78]	Observational case series	28 MS patients	Acupuncture induced spasms and clonus, proposed as an early MS symptom sign; treatment effects difficult to isolate due to spontaneous remission.
**Experimental Studies**			
Tang et al. (2025, China) [79]	Narrative Review	Review of acupuncture in neurodegenerative diseases	Acupuncture suppresses neuroinflammation, enhances autophagy, improves synaptic plasticity in diseases including MS.
Gamus & Shoenfeld (2025, Israel) [80]	Narrative Review	Review of clinical and experimental evidence in autoimmune diseases	Acupuncture modulates immune response and inflammation in autoimmune diseases including MS; analgesic effects noted.
Wang et al. (2025, China) [81]	Experimental animal study	C57BL/6 mice with experimental autoinmune encephalomyelitis (EAE)	Daily electroacupuncture at ST36 increased neural stem cell proliferation and differentiation into oligodendrocyte progenitor cells, improved clinical scores, reduced demyelination, and decreased CNS leukocyte infiltration. Effects were mediated through betaendorphin/ opioid signaling and were abolished by naloxone, supporting a mechanistic role for this pathway in remyelination.
Kim et al. (2012, South Korea) [82]	Experimental animal study	Rats with EAE	Qi acupuncture reduced paralysis incidence and duration, decreased inflammatory cells and CD4+ T cells in EAE rats versus controls.

Huang et al. (2011, China) [83]	Experimental animal study	Rats with ethidium bromide-induced demyelinated spinal cord	Electroacupuncture promoted oligodendrocyte precursor cells (OPC) proliferation and differentiation, increased remyelination and functional recovery.
**Reviews / Meta-Analyses of Acupuncture in MS**			
Chen et al. (2026, China) [84]	Systematic review and Bayesian network metaanalysis of RCTs	23 RCTs; 1,384 PwMS	Eight acupoint stimulation interventions were compared. Electroacupuncture plus acupoint injection showed the greatest improvement in disability and neurological function and activities of daily living (BI). Acupressure performed best for fatigue relief. Authors concluded findings are promising but exploratory because of methodological limitations and heterogeneity.
Chen et al. (2025, China) [85]	Systematic review and meta-analysis of RCTs	11 RCTs; 574 PwMS	Acupuncture combined with medication was more effective than medication alone for improving disability status and quality of life. Acupuncture related adverse events were rare and mild. Overall certainty of evidence was low due to substantial heterogeneity, so conclusions should be interpreted cautiously.
Chang et al. (2026, China) [86]	Systematic review and network meta-analysis of RCTs	73 RCTs; PwMS	Several non-pharmacological interventions significantly improved fatigue versus control, including exercise, cognitivebehavioral therapy, acupuncture or massage, eHealth, and combined interventions. Combined interventions appeared to be the most effective option for reducing fatigue in MS.
Haider et al. (2024, Pakistan) [87]	Systematic Review and Meta-analysis	6 studies (2 observational, 4 RCTs) of PwMS	Acupuncture significantly reduced fatigue and improved quality of life in MS patients; calls for higher quality trials.
Amatya et al. (2024, Australia) [7]	Systematic Review	32 RCTs, 1,481 Pw MS	Moderate to low-certainty evidence for physical activity and TMS in spasticity management; very low-certainty for acupuncture and others.
Moskvin et al. (2024, Russia) [88]	Literature Review	87 articles analyzed, clinical studies included	Low-level laser therapy is promising in MS treatment with mechanisms and study results presented; optimization parameters suggested.
Bapir et al. (2022, Multinational) [89]	Systematic Review & Meta-analysis	62 RCTs for neurogenic overactive bladder including MS	Moderate efficacy of various pharmacological and non-pharmacological therapies; acupuncture included but no clear superiority shown.
Kannan et al. (2022, HK/Nigeria) [90]	Systematic Review & Meta-analysis	23 RCTs on physiotherapy for central neuropathic pain including MS	Acupuncture reduces pain severity in MS and stroke; evidence supports acupuncture as part of pain management.
Wang et al. (2022, China) [91]	Literature Review	Review of studies on autoimmune diseases including MS	Acupuncture shows promising therapeutic effects by modulating immune responses in autoimmune diseases like MS.
Khodaie et al. (2022, Iran/China) [9]	Literature Review	Review of 31 acupuncture studies in MS	Acupuncture improves neuro-immune regulation, QoL, fatigue, bladder function, spasticity, pain, and may delay disease progression.
Zhu et al. (2019, China/HK) [8]	Narrative Review	Reviews studies on spasticity including MS, stroke, SCI, cerebral palsy	Moderate evidence electroacupuncture + routine care reduces spasticity after stroke; insufficient evidence for spasticity in MS.
Lin & Hsieh (2020, Taiwan) [92]	Literature Review	Review of clinical applications of bee venom acupuncture	Bee venom acupuncture shows anti-inflammatory and analgesic effects in MS and other neurological disorders.
Martens et al. (2017, Belgium) [93]	Systematic Review	18 studies on spasticity in disorders of consciousness including MS	Intrathecal baclofen & splints effective; acupuncture suggested but needing further study; overall spasticity poorly studied here.
Campbell et al. (2016, Scotland) [94]	Systematic review	13 RCTs including progressive MS patients	Physiotherapy interventions including acupuncture showed positive results in outcome measures; most studies underpowered; more research needed.
Karpatkin et al. (2014, USA) [95]	Narrative review	Review of 12 studies on acupuncture in MS	Many studies report improvements in fatigue, spasticity, pain, and QoL but are limited by poor design and lack of controls; stronger trials needed.
Hao & Hao (2012, USA) [96]	Narrative review	—	Chinese scalp acupuncture integrates traditional methods with western medicine; effective in acute and chronic CNS disorders with rapid clinical effects noted.
Bowling (2010, USA) [97]	Narrative review	—	Acupuncture considered low risk and possibly beneficial in MS; CAM widespread but evidence varies by modality.
Zhao & Li (2007, China) [98]	Clinical report/description	Not specified	Acupuncture and moxibustion at "eight acupoints at the head" combined with herbs applied in MS treatment and neurological disorders.
Lee et al. (2007, Korea) [99]	Narrative review	—	Reviewed acupuncture in neurological disorders including MS; concluded evidence is contradictory and more rigorous trials needed.
Brañas et al. (2000, UK) [100]	Systematic review	Literature-based	Reviewed treatments for MS-related fatigue including acupuncture; limited evidence for any treatment; amantadine showed some effect.
Spoerel et al. (1974, Canada) [101]	Letter/report	—	Early letter discussing acupuncture and MS; no new data presented.

Supplementary Table S4. Clinical and experimental and review studies of acupuncture in MS.

Abbreviations (Alphabetically): *BICAMS: Brief International Cognitive Assessment for Multiple Sclerosis; CAM: Complementary and Alternative Medicine; CD4+: Cluster of Differentiation 4 Positive; CNS: Central Nervous System; EAE: Experimental Autoimmune Encephalomyelitis; EDSS: Expanded Disability Status Scale; MS: Multiple Sclerosis; OPC: Oligodendrocyte Precursor Cells; PPAR-α: Peroxisome Proliferator-Activated Receptor Alpha; PwMS: People with Ms; QoL: Quality of Life; RCT: Randomized Controlled Trial; RRMS: Relapsing-Remitting Multiple Sclerosis; SCI: Spinal Cord Injury; TMS: Transcranial Magnetic Stimulation.*
